# Subjective Evaluation of Basic Emotions from Audio–Visual Data

**DOI:** 10.3390/s22134931

**Published:** 2022-06-29

**Authors:** Sudarsana Reddy Kadiri, Paavo Alku

**Affiliations:** Department of Signal Processing and Acoustics, Aalto University, Otakaari 3, FI-00076 Espoo, Finland; paavo.alku@aalto.fi

**Keywords:** naturalistic audio–visual emotion database, feature extraction, emotion analysis, emotion recognition, emotion synthesis

## Abstract

Understanding of the perception of emotions or affective states in humans is important to develop emotion-aware systems that work in realistic scenarios. In this paper, the perception of emotions in naturalistic human interaction (audio–visual data) is studied using perceptual evaluation. For this purpose, a naturalistic audio–visual emotion database collected from TV broadcasts such as soap-operas and movies, called the IIIT-H Audio–Visual Emotion (IIIT-H AVE) database, is used. The database consists of audio-alone, video-alone, and audio–visual data in English. Using data of all three modes, perceptual tests are conducted for four basic emotions (angry, happy, neutral, and sad) based on category labeling and for two dimensions, namely arousal (active or passive) and valence (positive or negative), based on dimensional labeling. The results indicated that the participants’ perception of emotions was remarkably different between the audio-alone, video-alone, and audio–video data. This finding emphasizes the importance of emotion-specific features compared to commonly used features in the development of emotion-aware systems.

## 1. Introduction

Emotions are natural phenomena in communication between humans. Emotions are what give communication life, and they make communication between humans bright and lively [1,2]. Communication without emotions is unnatural and apathetic and therefore difficult to participate for most of us for a long time. Emotions can be recognised automatically by computer using both visual [3,4,5,6] and audio [3,4,5] data, as well as their combination, audio–visual data [3,4,5,7]. Recognition of emotions from audio–visual data is effective for most real-life applications and can be conducted using data acquired with simple set-ups [8,9]. Motivated by a broad range of commercial applications, automatic emotion recognition has gained increasing research attention over the past few years. Automatic emotion recognition has been applied, for example, in call centers and clinics [10,11,12,13]. In call center services, an emotion recognition system can be used to assess customers’ satisfaction. In clinical environments, an emotion recognition system can help clinicians to access psychological disorders [14,15,16,17,18].

In order to build automatic emotion recognition systems and in order to analyse emotions, the primary requirement is to have access to an emotion database. Therefore, many research groups studying emotions have collected different databases from appropriate sources such as acting, induction, application-driven, and naturalistic sources [19,20,21]. Emotion databases published by different research groups can be categorized as simulated, semi-natural, and natural databases [19,20,22].

*Simulated emotion databases* are recorded from speakers/professional artists by prompting them to enact/pose emotions through specified texts. The main disadvantage is that deliberately enacted/posed emotions are quite deviant from spontaneous emotions, and they lack a proper context [21,23]. In [21], the main limitations in collecting emotion databases using acted emotions were discussed. The authors suggested guidelines for the design of corpora recorded from actors in order to reduce the gap between laboratory conditions and real-life applications. *Semi-natural emotion databases* are enacted/posed databases where a context is given to the speakers [24]. The third type of emotion databases are represented by *natural emotion databases,* where recordings do not involve any prompting or obvious eliciting/posing of emotional responses [25]. Typical data sources for natural emotion databases are television (TV) talk shows, interviews, podcasts, and group interactions [20,26]. The design and collection of an emotion database depends on the underlying application. For example, in studying synthesis of emotional speech, researchers typically collect emotional speech from a single talker or a few talkers [27,28,29,30] whereas in the area of speech emotion recognition, researchers typically collect data from multiple speakers. More details of the various types of databases, issues, and important aspects in emotion databases are described in [19,31].

As most of the real-world data available in social multimedia are in the audio–visual format, analysis of emotions in audio–visual data provides considerable advantages compared to the audio-alone or video-alone formats [4,5,8,9,32]. Therefore, in recent years, analysis of audio–visual data has had significant attention from many researchers. In general, the main benefit provided by processing audio–visual data instead of video-alone data is that video information might be occasionally poor due to recording quality, multiple persons in the video segment, movement of the speaker, etc. Hence, the use of audio–visual data has attracted increasing interest in studying automatic recognition of emotions in recent years. The literature of the study area shows that studies have been mainly carried out by selecting only good-quality data—where the underlying emotions are clearly expressed—for further analysis [7,33,34,35,36]. In addition, a few studies have focused on the perceptual evaluation of audio–visual emotion data to understand the perception of emotions [35,37,38,39,40,41,42].

In [39,40], authors conducted a perceptual evaluation to study whether emotional speech is real or acted in the valence dimension (i.e., positive or negative). They observed that acted emotions (especially negative emotions) were perceived more strongly than real ones hence questioning the usefulness of acted emotions. Moreover, the authors of [37,43,44] conducted perceptual tests for the recognition of various emotional expressions using visual imaging of speaker’s face in the valence dimension. It was observed that the recognition speed was faster for positive emotions than for negative emotions, and also that acted emotions were perceived as more intense than true emotions [43]. In [35], authors used spontaneous expressive mono-word utterances and the corresponding acted utterances (collected simultaneously) for perceptual evaluation with native French listeners in audio-only, visual-only, and audio–visual data. The aim in their study was to understand whether the listeners were able to discriminate acted emotions from spontaneous emotions. The results of their study indicate that listeners are indeed able to discriminate spontaneous emotions from simulated emotions. The perceptual evaluation test based on the audio–visual emotion data was conducted in [45] for two different cultures (Dutch and Pakistani speakers). Their study showed that acted emotions of speakers in both of the cultures were perceived as stronger than non-acted emotions. In addition, for the Dutch speakers, the negative emotions were perceived as relatively stronger, whereas for the Pakistani speakers, the positive emotions stood out perceptually. A more detailed analysis of facial expressions of emotions is described in [6,46]. In all these perceptual evaluation tests, the main focus was on understanding the difference between whether the data represents acted or real emotions, and the studies were conducted by selecting only good-quality emotional data for analysis.

The general goal of this paper is to understand human perceptual patterns (cues) in the perception of emotions in natural emotion data. Perceptual evaluation tests are conducted using three modes of natural emotion data, namely audio-alone, video-alone, and audio–visual data. It is to be noted that the data of the audio-alone and video-alone formats are taken from tracks of audio–visual data. In addition, we aim to understand the contribution of each modality for developing emotion-aware systems.

The organization of the paper is as follows: Section 2 describes the naturalistic emotion database used in the perceptual evaluation. Section 3 describes the perceptual evaluation procedure. Results and discussion are given in Section 4 and Section 5, respectively. Finally, Section 6 draws conclusions of the study and discusses scopes for further studies.

## 2. Database

The International Institute of Information Technology-Hyderabad (IIIT-H) audio–visual emotion database (IIIT-H AVE) was chosen for the current study as a source of natural audio–visual emotion data [47]. This database has been collected from English movies and soap-operas from TV broadcasts. The database includes data in the audio-alone, video-alone, and audio–visual modes. The data clips were manually annotated using two labeling approaches (categorical and dimensional). In the categorical approach, the data was labeled into seven basic emotions (angry, disgusted, frightened, happy, neutral, sad, and surprised) and six expressive states (confused, excited, interested, relaxed, sarcastic, and worried) [19,48]. The list of emotions and expressive states are shown in Table 1.

In the dimensional approach, the database was labeled in two dimensions, namely arousal (active or passive) and valence (positive or negative). The criteria adopted for selecting ‘a good source clip’ were the following:

- *Audio–visual clips with no background music or noise.*
- *Clips with only one speaker speaking at a time.*

**Figure 1:**
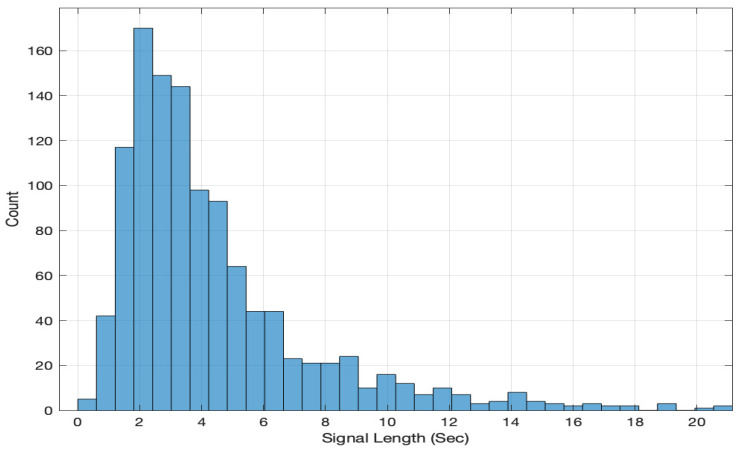
Histogram for the signal length (duration).

For the present study, four basic emotions (angry, happy, neutral, and sad) in the categorical approach and two dimensions (arousal and valence) in the dimensional approach are considered. For the categorical approach, the confidence scores (ranging from 2 to 9, where 2 corresponds to the lowest confidence and 9 corresponds to the highest confidence) were specified because emotions are expressed on a continuum in natural data. The confidence of 1 corresponds to neutral. The database consists of 1176 labeled clips, of which 741 clips are from male speakers and 435 clips are from female speakers in all the three modes. The statistics of data as per emotion and per speaker is given in Tables 2 and 3, respectively, in [49]. The histogram of the signal length (duration) is shown in Figure 1. The video files are MPEG4-coded image sequences, mostly with frames of 1280 × 720 pixels. All the extracted audio wave files have sampling rates of 44.1 kHz or 48 kHz and are either in the stereo or mono format. The audio data were down-sampled to 16 kHz and expressed in the mono format. The IIIT-H AVE database is publicly available [49].

## 3. Perceptual Tests

The aim of this study is to understand the perception of emotions in humans in terms of perceptual cues or patterns. Specifically, the study focuses on perceptual cues and their relation between auditory and visual cues in different emotions, and on the differences between audio-alone and video-alone data in perception of emotions. For this purpose, perceptual tests were carried out using the three data modes and the two labeling approaches.

### 3.1. Participants

Ten participants (five male and five female) took part in the perceptual tests. All of the participants were students and research scholars of the IIIT-Hyderabad. The mean age of the participants was 23 years (ranging from 20 to 25). None of the participants were involved in the collection of the IIIT-H AVE database. The mother language of all the participants was Telugu (one of the major Indian languages) and their second language was English.

### 3.2. Procedure

The participants were assigned to evaluate all three modes of the data. Two sets of perceptual evaluations were made, the first using the data labeled with the categorical approach and the second using the data labeled with the dimensional approach. A part of the entire IIIT-H AVE database was chosen for this purpose. For the study of categorical labeling, 50 emotion clips were selected for each of the four basic emotions (angry, happy, neutral, and sad) which consisted of confidence scores in the range from 5 to 9 in nearly equal distribution. These four basic emotions are considered to be universally recognizable emotions. Many speech emotion recognition studies (e.g., [4,24,50,51,52,53]) have been conducted using these basic emotions, and therefore they were also studied in the current paper. In the perceptual evaluation of the dimensional approach, the corresponding emotion clips were rated for the following four combinations: AP (active-positive), AN (active-negative), PP (passive-positive), and PN (passive-negative), and the neutral samples were labeled as NN (neutral-neutral).

The evaluation was carried out first using the audio-alone data, then using the video alone data, and finally using the audio–visual data. The participants were asked to recognize the presented emotion and its dimension. The emotion samples were randomized and participants could choose any emotion category (i.e., participants were not asked to choose one among the four emotions in the categorical approach). Participants were asked to judge the emotion or expressions or any other category based on their perception. The audio samples were presented using headphones. The participants were trained with five audio–visual files for each emotion. If the participant identified 90% of the files correctly, he/she was allowed to do the perceptual tests. However, the participants were allowed to hear the samples as many times as they wished. In addition, the participants were allowed to take breaks (5 to 10 min) if they wished.

After the perceptual evaluation of the audio-alone, video-alone, and audio–visual data, the participants were asked several questions by the first author. The questions were selected by first studying the responses of the participants (emotion ratings and descriptions). The participants were asked the following main questions. (Note that all the questions below were asked after the entire session and not after every utterance.)

- What is your order of preference in the judgment of emotion between the audio-alone, video-alone, and audio–visual data?
- Is the entire dialogue needed for the perception of the emotion?
- How can you describe each emotion category that you specified in the evaluation sheet based on the audio-alone, video-alone, and audio–visual data?
- What are the auditory and visual cues that you focused on in the judgment of emotions?
- What are the difficulties in the judgment of emotions in all three data modes?
- How much focus do you give to audio (without linguistic content) to judge a particular emotion?
- How much focus do you give to the linguistic content?
- Which pair of emotions is confusing? Why? How are you judging it?
- How do you discriminate angry and happy, neutral and sad, worried and sad?
- If multiple emotions occur, what are they? How do they occur (in sequence, for example) and how do you decide?
- How do you identify emotions if video quality is not good or person is not visible completely?
- Do you identify emotions based on body postures, gestures, and side view, etc.?
- Is your judgement based on other persons’ reactions in the video-alone and audio–visual data?
- If you listen/watch more than once, why you do it? How do you decide emotions in this case?
- Do you identify any audio–visual samples that show face as one emotion and audio as different emotion?

In total, the test took around one hour for each participant, including the breaks and around thirty minutes for the question and answer session.

## 4. Results

The perceptual cues may help in understanding the human way of processing emotions and this knowledge can be used in developing emotion-aware systems. In this section, the findings of the perceptual evaluation in the categorical approach and dimensional approach are described. Table 2 reports the order of preference based on the highest recognition of emotions from the audio–visual data (‘***’ means the highest preference and ‘*’ means the lowest preference). From the table, it can be seen that the recognition of angry is more prominent in the audio-alone data than in the video-alone or audio–visual data. However, happiness is easily recognizable from the video-alone data, and sadness is recognizable better from the audio–visual data.

Table 3, Table 4 and Table 5 report the identified emotions from the audio-alone, video-alone, and audio–visual data, respectively. From Table 3 (audio-alone), it can be observed that there is confusion between neutral and sad. In addition, sometimes sad is recognized as worried, happy is recognized as surprised, and angry is recognized as excited. In the video-alone data (Table 4), recognition of happy is an easier task, and happiness is recognized as surprise sometimes. From the audio–visual data (Table 5), it can be observed that there is not much confusion among the emotions except sadness is sometimes recognized as worried.

## 5. Discussion

In the perceptual experiments, it was observed that angry utterances are perceived as shouting, frustrated, disgusted, and excited, etc., and these can be considered as variants of angry or higher activation states. Similarly happiness was sometimes identified as laughter, smiling, excited, and surprised (sometimes using lexical information), whereas sadness was sometimes identified as worried, boredom, calm, and sorrow, etc., which are variants in passive or low-activation states. These variants can be considered as expressive states rather than emotions. The distinction between these two terms is made according to the amount of time conveyed (i.e., unsustainable/short time for emotion and sustainable/longer time for expression).

**Figure 2:**
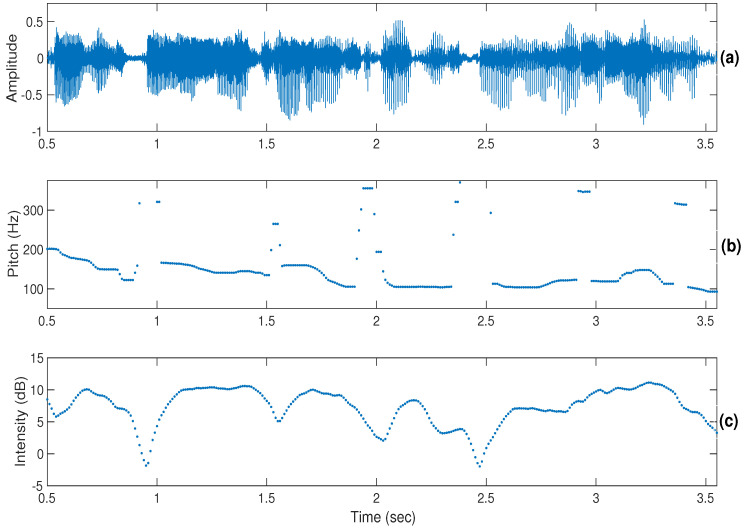
Illustrations of the pitch (shown in (**b**)) and intensity (shown in (**c**)) contours of a speech signal (shown in (**a**)) in neutral emotion.

**Figure 3:**
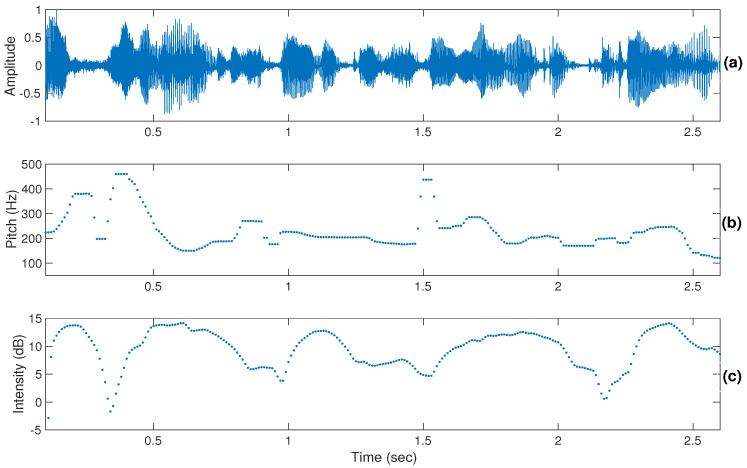
Illustrations of the pitch (shown in (**b**)) and intensity (shown in (**c**)) contours of a speech signal (shown in (**a**)) in high-arousal angry emotion.

**Figure 4:**
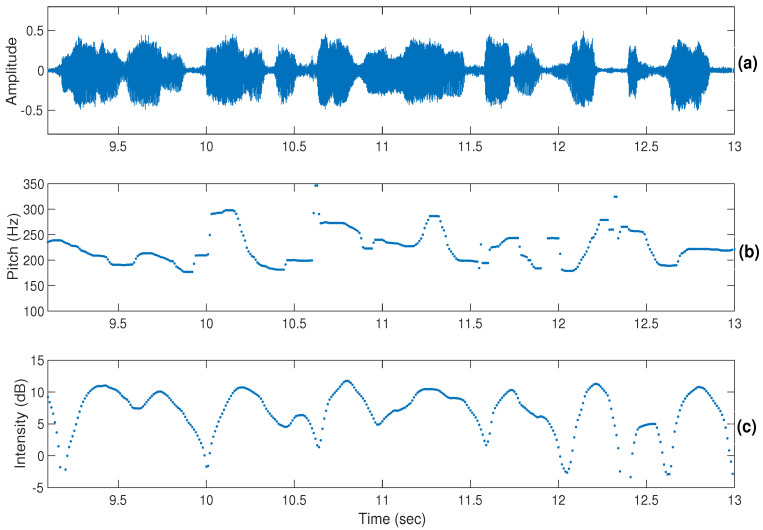
Illustrations of the pitch (shown in (**b**)) and intensity (shown in (**c**)) contours of a speech signal (shown in (**a**)) in high-arousal happy emotion.

For the dimensional approach, it was observed that the discrimination of emotions was easier than in the categorical approach. In the dimensional approach too, discrimination of activation (active/passive) was easier than valence (positive/negative). It was found that the participants sometimes used lexical information in discriminating positive or negative emotions. The perceptual cues for the discrimination of activation were mainly pitch, intensity, and loudness, whereas in the valence discrimination, the temporal patterns along with the linguistic content information played the main role. The discrimination of activation is possible even from dialogues of short duration, whereas in the discrimination of valence it is not an easy task, and participants often use lexical information in discriminating valence. Figure 2, Figure 3 and Figure 4 show illustrations of pitch and intensity contours [53] for neutral, angry, and happy emotions, respectively. From the figures, it can be observed that pitch and intensity values for angry (Figure 3) and happy (Figure 4) emotions are higher with more variations within an utterance compared to neutral (Figure 2).

It was observed that humans can easily discriminate dimensional-wise emotions independently of the language/lexical content. On the other hand, the perception of emotions in the categorical approach is sometimes dependent on the lexical content and also varies between emotions in audio-alone and audio–visual data. The following issues are the most important observations made from the participants’ answers to the various questions.

- It was observed that the entire duration of the speech/video may not show emotions properly, but emotions are well-represented only by some segments of the dialogue.
- Emotions are unsustainable and the speaker can not be in an emotional state for a long time. Thus, only some segments contribute to the perception of emotions, while the remaining segments appear to be similar to non-emotional normal speech. Hence, the identification of emotional segments in an entire dialogue is a justified research problem to work on.
- There are different types of temporal prosody patterns (and voice quality variations) for different emotions, and they have a prominent role compared to lexical information.
- For full-blown emotions (such as angry and happy), the identification of emotions is a easier task. The use of lexical information increases the discrimination confidence.
- In most cases, identification of negative emotions takes relatively more time compared to positive emotions both in the audio and video data.
- An angry voice seems to be more intelligible with some creakiness or harshness, whereas in happy voices breathiness (noisy structure), laughter, and some temporal patterns such as rhythm with pleasant voice are present.
- Anger typically shows a sudden change of intensity in a very short period of time. With happiness, intensity rises slowly, but it can stay at a high level for a longer time compared to anger.
- In general, humans can easily perceive emotions from clips of longer duration. This implies that temporal information enables more rapid perception of emotions compared to segments of a shorter duration and it depends on the emotion category.
- Participants listened to/watched some samples more than once if mixed or multiple emotions occurred in them, or if the samples were expressive and therefore difficult to categorize, or when the length of the sample was short.
- Some of the multiple emotion combinations are: excited followed by angry, angry followed by frustrated or disgusted, sad followed by worried, and excited followed by shouting (mostly in angry).
- In the video-alone and audio–video data, the participants perceived emotions not only from actors’ faces but also from their body gestures, or, for example, based on other persons’ reaction in video. Other persons’ reactions also sometimes created confusion in identifying emotions.

**Figure 5:**
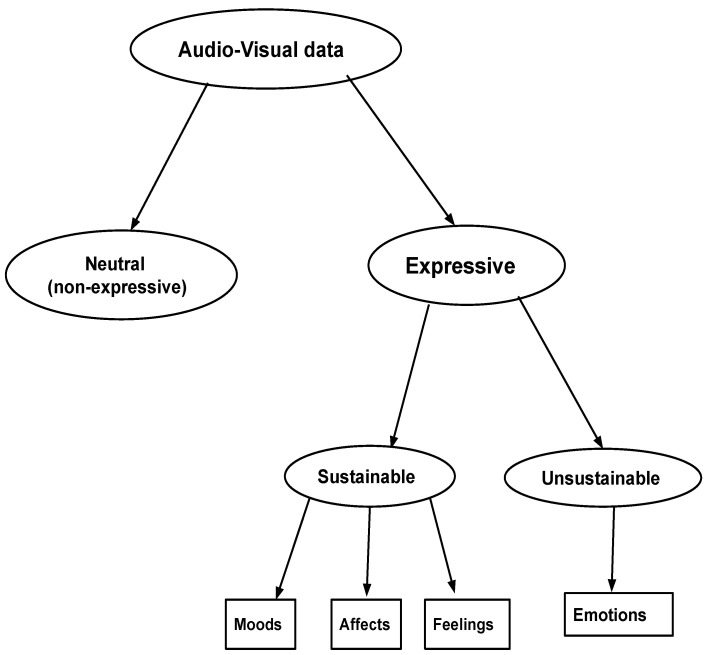
An hierarchical approach for analyzing audio–visual data.

Overall, the study showed that, in general, the role of temporal information is important in the perception of emotions by humans from audio and video data. Initially, humans seem to distinguish whether the data correspond to expressive or non-expressive speech, and then whether the data correspond to a sustained or non-sustained state. The sustained state has lower intensity and it maintains for a longer time, whereas the non-sustained state has higher intensity and cannot be maintained for a long time. Based on this, a hierarchical approach is shown in Figure 5. In this approach, an initial hierarchical decision is made first based on whether the audio–visual data is expressive or non-expressive (neutral). Furthermore, the expressive data is divided based on whether it is sustainable or not. If the data is sustainable, it is assigned to carry moods, feelings, affects, etc., if not, the date is assigned to carry emotions. This hierarchical tree (Figure 5) is also in conformity with the tree-like structure reported in [53] (see Figure 1.1 on Page 4 in [53]).

In summary, in the present study perceptual evaluations were carried out for the recognition of emotions using the categorical approach and the dimensional approach. Results show that different emotion-specific features need to be explored for the development of emotion-aware systems. In addition, it was observed that in order to gain a higher confidence level in the recognition of emotions, the combination of audio and video data is needed. This is also in line with the studies reported in [4,5,54,55,56,57,58,59,60]. For some emotions (such as angry) audio cues play a more important role, whereas for some other emotions (such as happy) video cues are more important and the combination of the both cues improves the confidence in emotion recognition.

## 6. Conclusions

Even though the present study investigated the perceptual evaluation of four emotions, a larger number of emotions should be studied in the future using the same audio–visual approach. Although the database used in the current study is a naturalistic emotion database, collecting more realistic data is still needed to cover wider dynamics of human communication in terms of lexical contents, languages, environments, culture, etc. Moreover, instead of relying on clips from TV broadcasts, as in the present study, studying emotions based on natural human conversation data also enables self-reported collected emotions to be used as the ground truth in perceptual evaluations. In addition, the current perceptual evaluations can be extended by recruiting also such participants to the evaluations who cannot understand the language used in audio–visual data. This would demonstrate the role of lexical information in the perception of emotions. The mean age of the participants in this study was 23 years (ranging from 20 to 25). As the perception of emotions depends on age, and as the participants of the present investigation were all quite young, the results of this study should not be generalized to people of all ages. In addition, the mother tongue of the participants (Telugu) was different from the language of the recorded data (English), and this language mismatch may have affected the overall results of the experiments. These issues need to be considered in further investigations of the topic.

It was found that only some segments in the entire dialogue/video were taken advantage of in the identification of emotions, and hence a method of detecting such unsustainable regions is a good research problem to be investigated in the future. Judging mixed/multiple emotions is ambiguous, and it appears that temporal information plays a prominent role in identifying emotions. Hence, the identification of emotions in the current segment might benefit from the identification decision of the previous segments in the development of automatic emotion recognition systems. As many speech emotion recognition studies have reported confusion between the recognition of angry and happy, developing better perceptual features for these two emotions should improve emotion recognition systems. In addition, these better emotion-specific features might be helpful in the discerning between acted emotions and real ones. The results of this study suggest that utterances with shorter duration or short segments within an entire utterance are responsible for better recognition of high arousal emotions. Hence, these issues should be considered in aiming at improved performance of emotion recognition systems [57].

## Figures and Tables

**Table 1:** List of emotions and expressive states.

Emotions	Expressive States
1. Angry	1. Confused
2. Disgusted	2. Excited
3. Frightened	3. Interested
4. Happy	4. Relaxed
5. Neutral	5. Sarcastic
6. Sad	6. Worried
7. Surprised	

**Table 2:** Order of preference in the perception of emotions with respect to neutral. The notation ‘***’ refers to the highest preference and the notation ‘**’ refers to the medium preference and ‘*’ refers to the lowest preference.

	Audio-Alone	Video-Alone	Audio–Video
Angry	***	*	**
Happy	**	***	**
Sad	*	**	***

**Table 3:** Identified affective states (denoted by *) for the four emotions in the audio-alone data.

	Angry	Happy	Neutral	Sad	Excited	Worried	Surprised
Angry	*	-	-	-	*	-	-
Happy	-	*	-	-	-	-	*
Neutral	-	-	*	*	-	-	-
Sad	-	-	*	*	-	*	-

**Table 4:** Identified affective states (denoted by *) for the four emotions in the video-alone data.

	Angry	Happy	Neutral	Sad	Excited	Worried	Surprised
Angry	*	-	-	-	*	-	-
Happy	-	*	-	-	-	-	*
Neutral	-	-	*	*	-	-	-
Sad	-	-	-	*	-	*	-

**Table 5:** Identified affective states (denoted by *) for the four emotions in the audio–video data.

	Angry	Happy	Neutral	Sad	Excited	Worried	Surprised
Angry	*	-	-	-	*	-	-
Happy	-	*	-	-	-	-	*
Neutral	-	-	*	-	-	-	-
Sad	-	-	-	*	-	*	-

## Data Availability

Database is publicly available at https://github.com/SudarsanaKadiri (accessed on 6 May 2022).
